# Ocular Translation Due to Gravitational Acceleration

**DOI:** 10.1167/iovs.66.14.21

**Published:** 2025-11-12

**Authors:** Joseph L. Demer, Robert A. Clark

**Affiliations:** 1 Department of Ophthalmology, University of California, Los Angeles, California, United States; 2 Stein Eye Institute, University of California, Los Angeles, California, United States; 3 Bioengineering Department, University of California, Los Angeles, California, United States; 4 Neuroscience Interdepartmental Program, University of California, Los Angeles, California, United States; 5 Department of Neurology, University of California, Los Angeles, California, United States

**Keywords:** head tilt, magnetic resonance imaging, optic nerve, translation

## Abstract

**Purpose:**

We asked if human globe position in the orbit is influenced by gravitational head orientation.

**Methods:**

In 11 adult volunteers, surface coil magnetic resonance imaging (MRI) in 2 mm thick axial and quasi-coronal planes with a centered visual target was performed in supine position that aligned the cranial midline along the gravity vector, and was repeated in right and left decubitus positions that oriented gravity orthogonally. From coronal MRI, we computed globe centroid positions relative to the whole head.

**Results:**

In decubitus posture relative to supine, the upper eye shifted 0.85 ± 0.55 mm anteriorly and 0.44 ± 0.49 mm medially (*P* < 0.005 for both), without shifting superiorly. The lower eye translated anteriorly by 0.73 ± 0.59 mm (*P* < 10^−^^4^), 0.36 ± 0.56 mm laterally (*P* < 0.02), and 0.25 ± 0.50 mm superiorly (*P* < 0.03). Vector magnitude of translation was significantly non-zero at 1.13 ± 0.55 mm for upper eyes and 1.16 ± 0.59 mm for lower eyes (*P* < 10^−^^7^).

**Conclusions:**

In supine position, gravity causes enophthalmos: a 0.73 to 0.85 mm shift in anteroposterior eye position that is significant relative to intraorbital optic nerve length. When in lateral decubitus position, the higher (upward) eye shifts nasally and anteriorly, so its translation is oblique to the orbital axis. The lower (downward) tilted eye shifts temporally and anteriorly along the orbital axis. Thus, the lower eye translation elongates the nerve by about threefold more than for the upper eye. Gravity therefore has an effect on the human eye that should be considered when modeling the effect of eye rotation on the mechanical state of the optic nerve and other orbital tissues.

The human eye is suspended in its bony orbit by an array of connective tissues that includes the pulleys of the extraocular muscles (EOMs), as well as a cushion of orbital fat. Although the most familiar eye movements are rotations, high resolution magnetic resonance imaging (MRI) has demonstrated that the eye also translates slightly during rotations in a manner that influences the leverage exerted by the extraocular muscles.^1^^,^^2^ This results in a to-and-fro rolling motion during horizontal eye rotations without anteroposterior translation. However, when the optic nerve (ON) becomes stiffened in primary open angle glaucoma, tethering by the inelastic optic nerve causes globe retraction during large angle adduction.^3^^,^^4^

Eye translation data available to date from MRI have all been obtained from subjects lying supine in MRI scanners. In this postural state, gravitational acceleration tends to move the globe posteriorly in the head and thus toward the orbital apex. Reaction forces from the passive tissues and oblique EOMs oppose the posteriorly directed forces of the rectus EOMs, in addition to gravitational acceleration acting on the mass of the globe, to situate the eye in mechanical equilibrium. Of course, gravitational acceleration does not act in the same way when the head is upright, such as in the standing or sitting positions typical of the human alert state. In the upright posture, gravitational acceleration no longer acts in the direction of retracting the globe toward the orbital apex. In the usual terrestrial situation, this creates a reduction in posterior force corresponding to mass of the globe of about 7.5 gram-force, an amount comparable to that of human lateral rectus muscle tension in central gaze.^5^ It is thus obvious that, because of the effect of gravity, the mechanical state of the orbit as studied by MRI in the supine position is not identical to the mechanical state in the upright position or during spaceflight. Astronauts returning from extended exposure to microgravity in earth orbit exhibit elongation of their ONs,^6^ suggesting that release of gravitational force on the globe may allow it to shift in the orbit.

The presence of gravity is unavoidable in terrestrial environments where MRI can practically be performed. The direction of gravitational acceleration relative to the orbit can, however, be manipulated by changing posture in an MRI scanner. For example, lateral decubitus positioning places the gravity vector perpendicular to the long orbital axis, so that gravity no longer tends to displace the globe toward the orbital apex. The current study employed this approach for a novel characterization of the effect of gravity on globe position within the orbit.

## Methods

Subjects were 14 healthy adult volunteers who consented in writing according to the tenets of the Declaration of Helsinki and approved by the Human Subject Protection Committee at the University of California, Los Angeles. No subject had a history or symptoms of any ophthalmic or neurological disease. Examination verified normal visual acuity, ocular motility, stereoacuity, and ocular anatomy.

**Figure 1.**
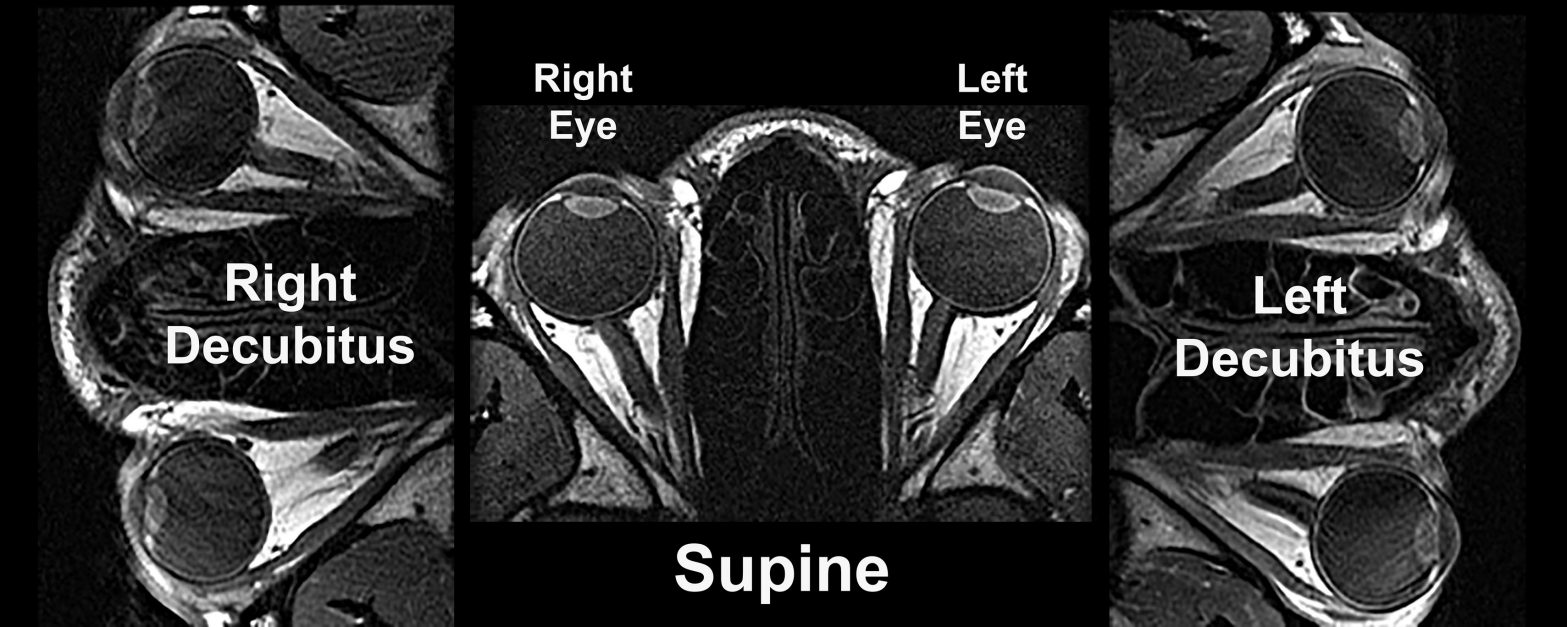
Axial T1-weighted gadolinium contrast magnetic resonance images of a normal subject in central gaze in supine and lateral decubitus positions.

High-resolution, T1 or T2 fast spin echo MRI was performed using a SIGNA 1.5T MRI scanner (GE HealthCare, Chicago, IL, USA) as described elsewhere,^7^^,^^8^ including use of a dual-phased surface-coil array (Medical Advances, Milwaukee, WI, USA) and central fixation targets. Some T1 studies involved intravenous gadodiamide contrast infusion. Subjects were scanned in supine position, as well as in 90° right side down and left side down decubitus positions (Fig. 1). Decubitus positioning was supported by stacks of linen sheets placed under the lower ear and side of the head. The head was kept close to magnet isocenter for all postures. During imaging, the scanned eye monocularly fixated a fine optical fiber illuminated by a light emitting diode in what was intended to be straight ahead gaze about 2 cm distant. However, small changes in target and head position were difficult to detect in the MRI scanner until imaging had been performed, so some images were inadvertently obtained in modestly eccentric lateral gazes. Studies with significant gaze eccentricity were excluded from final analysis.

A triplanar localizer MRI scan was first obtained to verify head positioning in each decubitus position, with correction of head orientation and repetition of the localizer MRI as necessary to achieve desired head orientation relative to gravity. Sets of 18 to 20 contiguous, 2-mm-thick quasi-coronal images were oriented perpendicular to the long axis of each orbit using a 256 × 256 matrix over an 8-cm field of view, yielding 312-µm resolution within the plane. Supine scans were obtained first and then right before the left decubitus posture. The right orbit was scanned before left orbit.

Analysis was initially by robust methods previously published. Images were analyzed using ImageJ (64-bit; National Institutes of Health, Bethesda, MD, USA). Quasi-coronal images of the left orbit were digitally reflected to the configuration of a right orbit. In these images, the globe and orbital walls were manually outlined.^9^^–^^11^ In axial images including the central lens and optic nerve from the angle, gaze direction was determined relative to midsagittal of a line passing from lens to the estimated foveal location, as has been found reliable elsewhere.^12^^,^^13^ For analysis of quasi-coronal MRI, a Cartesian orbital coordinate system was taken as positive in the lateral, superior, and anterior directions anchored at the orbital area centroid in the image plane containing the globe–optic nerve junction in supine position. The center of the best sphere fitting the globe was determined in scanner coordinates using cross-sectional images of the globe in three different planes^11^ for each head position. This approach determined the three-dimensional (3D) globe center from many voxels and so achieved resolution much better than the size of any one voxel. Then, anteroposterior locations of the globe centers in decubitus positions were registered to the orbital center in supine position by mapping the orbital cross-sectional area at the globe–optic nerve junction in those image sets to a best-fit line of orbital cross-sectional areas near the globe–optic nerve junction in the supine position. Again, this approach determined the 3D orbit center from many voxels and so achieved resolution much better than the size of any one voxel. Magnitudes of globe translation were computed directly from the differences in the 3D globe centers in decubitus positions compared with the supine position. This approach minimized potential effects of any long distance magnetic field non-uniformity, as it involves local distance changes between the closely adjacent globe and orbital centers.

Translations are independent of the coordinate system used; however, it is intuitively helpful to specify translations relative to familiar external anatomical references for the head. The lateral angle of each orbit relative to the cranial mid-sagittal line was measured for each orbit using ImageJ from axial images such as illustrated in Figure 1. This angle was used to geometrically transform the measured orbital coordinates to the true cranial coordinates reported here, although angular transformation introduced small roundoff errors that did not alter the conclusions of this study. Parametric statistical analyses were performed, including paired two-tailed tests and two-way analysis of variance. Each individual orbit was considered the unit of analysis. Every data point is presented graphically.

## Results

Subjects were 12 women and two men with average age 24 ± 4 years (range, 20–34). However, data were excluded from two women and one man due to insufficient centration of eye position in at least one decubitus gaze position. Notwithstanding this technical deficiency, none of the quantitative conclusions was changed by this exclusion. Interpretable data were obtained in 20 orbits of 11 subjects. In these included subjects, mean horizontal eye orientation in the upward oriented eye was 3.8° ± 5.3° abduction and in the downward oriented eye was 1.6° ± 5.3° adduction. Mean age of the included subjects was 24 ± 5 years. Mean maximum horizontal globe diameter of the included subjects was 25.0 ± 1.0 mm. Analysis of axial MRI indicated that, on average, the orbits were oriented 22.9° ± 2.8° lateral to the mid-sagittal line; however, the orientation of each individual orbit was used to compute translation of that eye relative to overall head position.

**Figure 2.**
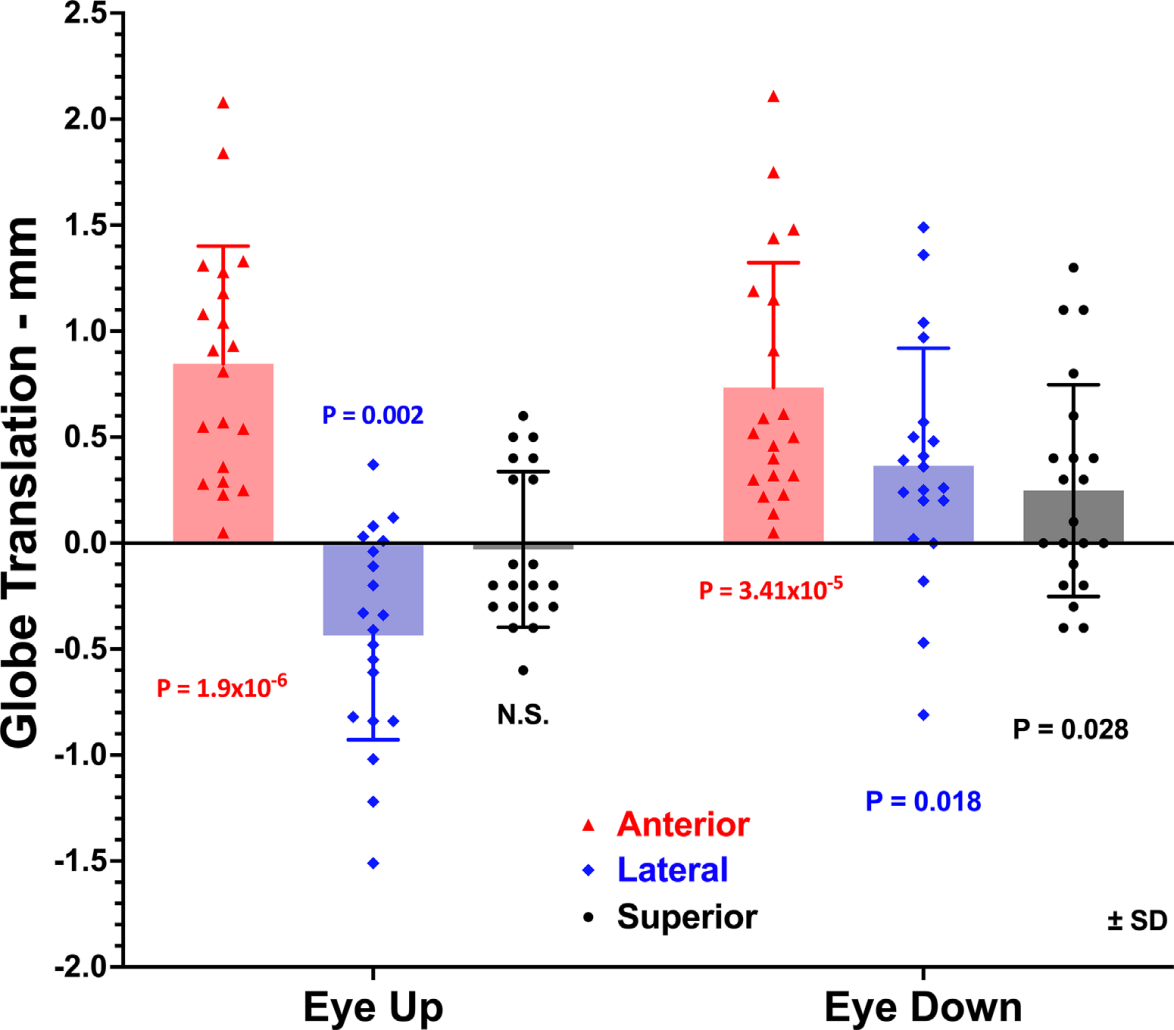
Mean globe translation from supine to decubitus positions, relative to the whole head. Each symbol represents one individual measurement. *P* values are for differences from zero.

Relative to supine, in the lateral decubitus position, in the cranial coordinate system the upper eye translated on average 0.44 ± 0.49 mm medially and 0.85 ± 0.55 mm anteriorly, as illustrated in Figure 2. Both translations differed significantly from zero (*P* < 0.02). There was no significant vertical translation by the upper eye. The lower eye translated anteriorly by a significant average of 0.73 ± 0.59 mm (*P* < 10^−^^4^), laterally by 0.36 ± 0.56 mm (*P* < 0.02), and superiorly by 0.25 ± 0.50 mm (*P* < 0.03).

It is informative to examine the behavior of individual cases represented by distinct symbols in Figure 2. Eye translation in decubitus positions was consistently anterior for all orbits oriented both upward and downward. Superior translation was small and randomly distributed about zero for the upper eye and for the lower eye was distributed about a small positive value. However, three orbits each in the upward and downward postures exhibited lateral translation opposite the average group behavior (Fig. 2). In total, this encompassed five subjects. In only one orbit of one subject was there mediolateral translation opposite the group behavior in both postures, but in this subject translation was typical in the fellow eye. In four other individuals, there was mediolateral translation opposite the group behavior in only one of the two orbits. Horizontal rotational eye orientation was as well controlled in all subjects exhibiting atypical translation as in the remaining subjects.

**Figure 3.**
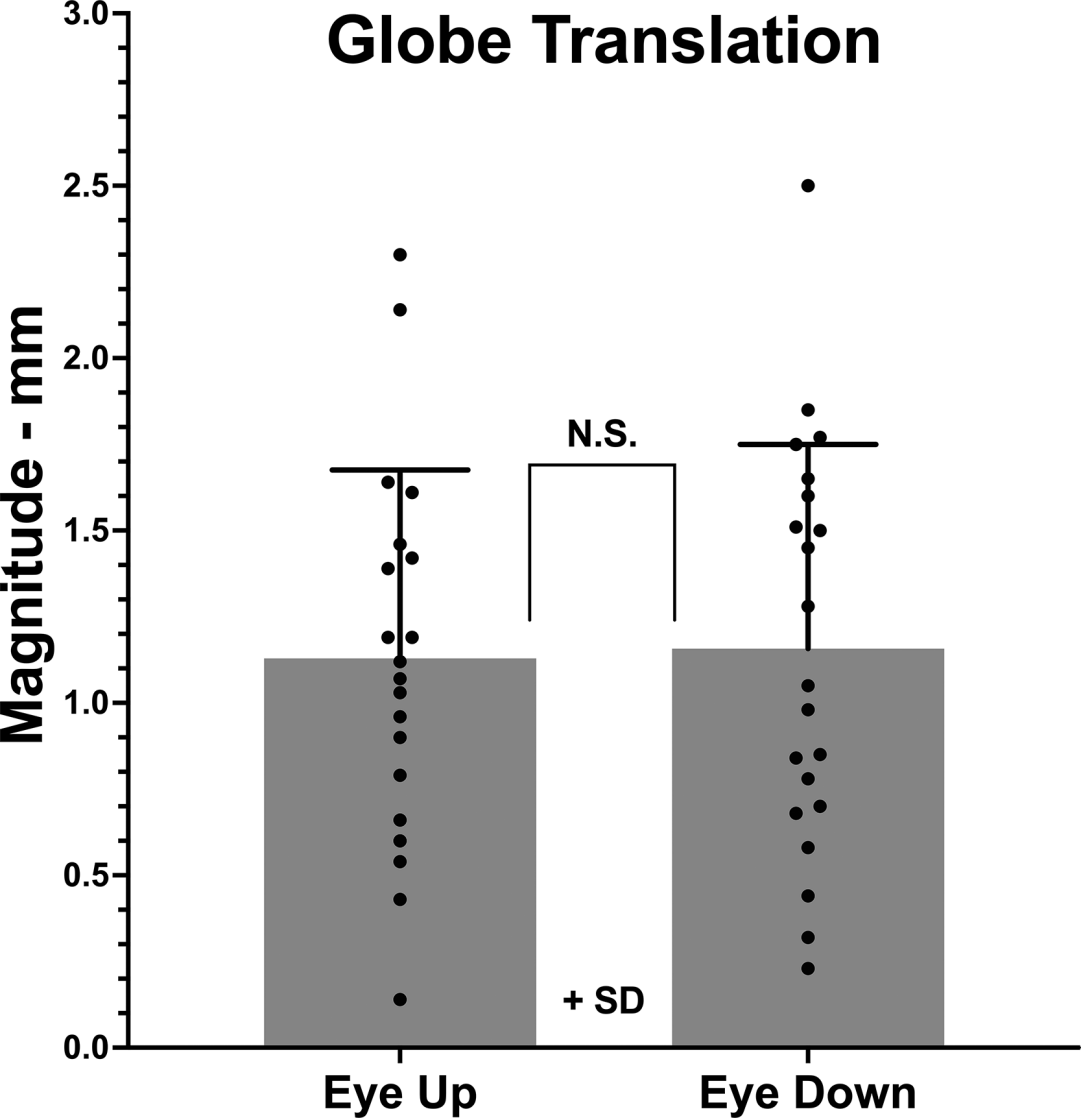
Mean globe translation magnitude; N.S., not significant.

**Figure 4.**
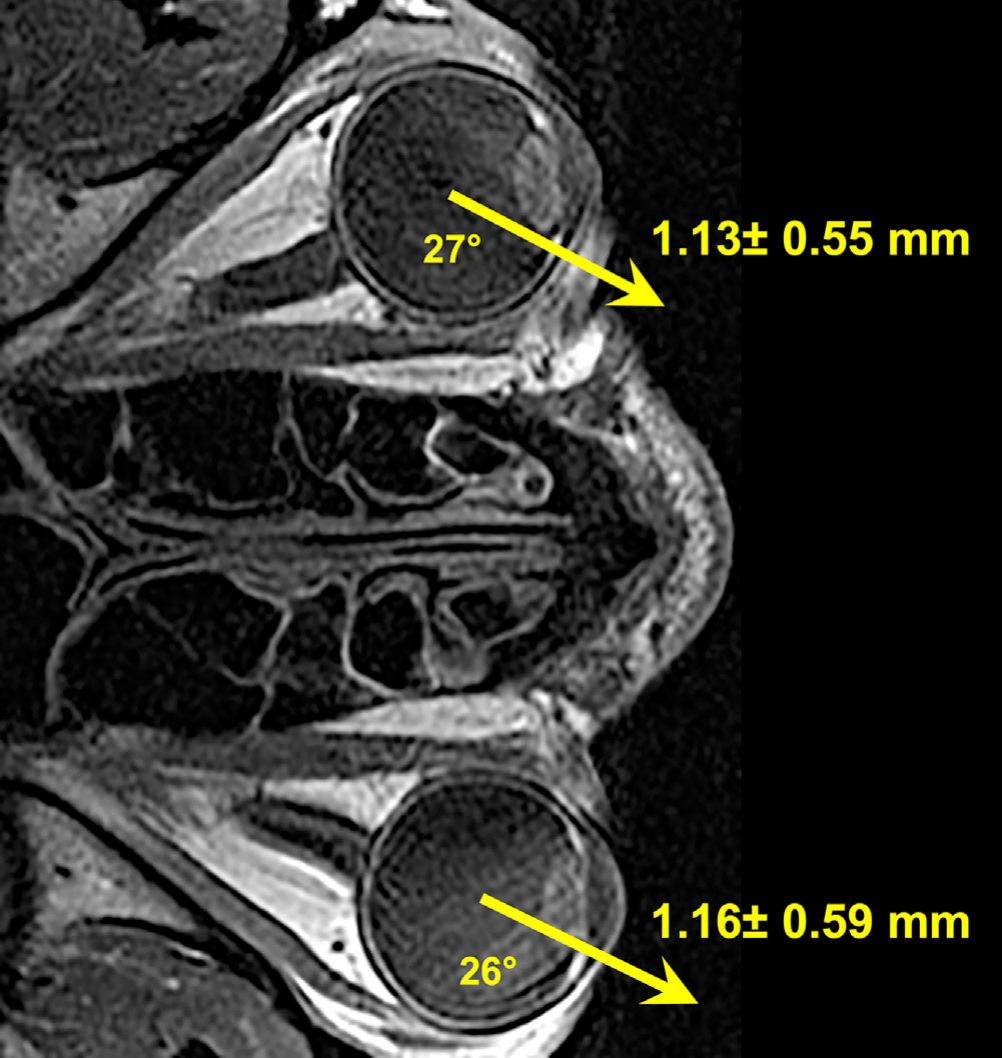
Summary of mean ± standard deviation of globe translation from a reference supine position to a lateral decubitus position, as superimposed on a representative axial T2-weighted MRI image. *Arrows* depict average angles from midsagittal plane. Data are from 20 orbits.

Including components in all three orthogonal directions relative to the supine reference position, translation magnitudes in decubitus position were 1.13 ± 0.55 mm in the upper eye and 1.16 ± 0.59 mm in the lower eye (Fig. 3). Being nearly identical, these magnitudes do not differ significantly. The translation of both eyes is summarized in Figure 4, a pictorial summary in the axial perspective of globe translation due to lateral head tilting. It is notable that both eyes shifted anteriorly and downward in approximately parallel directions relative to gravity. In the lower eye, this direction of translation was very close to alignment with the long axis of its orbit, whereas the upper eye translated both nasally and anteriorly, at an angle of about 72° to the long orbital axis.

## Discussion

Although it has been long recognized that the eye translates during rotation,^1^^,^^2^^,^^14^^–^^16^ we report here the novel finding that the human eye translates in response to decubitus head tilting relative to gravity. Compared with upright posture, in the supine position gravity causes 0.73 to 0.85 mm enophthalmos. Quantitatively similar to translation during horizontal eye rotation,^16^ the overall magnitude of translation from a supine to decubitus position is a little over 1.1 mm, but the direction of translation depends on whether the orbit is oriented upward or downward relative to gravity (Fig. 4). It is notable that the upward tilted eye shifts nasally as well as anteriorly about 72° to the long orbital axis, so that, by trigonometric projection, only 32% of the translation is along the orbital axis. In contrast, the downward tilted eye shifts temporally as well as anteriorly, almost exactly paralleling the orbital axis. This means that, during lateral decubitus tilting, the lower eye translates almost parallel to the ON, in a direction that would tend to elongate the nerve by about threefold more than is the case for the upper eye.

While globe translation in the lateral decubitus position was consistently anterior in all orbits, mediolateral translation from supine to lateral decubitus position was on average in the direction of gravity, so that the upper eye translated medially and the lower eye laterally. However, there were several individual exceptions to this behavior, with three eyes translating mediolaterally opposite the direction of gravity (Fig. 2). Given the interindividual variation in translation, some of this apparently paradoxical behavior may simply be the result of experimental noise. It may also be that mediolateral globe translation actually varies in direction among subjects and orbits in individual cases. This question would benefit from future study.

The approximately 1 mm change in globe position from supine to lateral head tilt is anatomically significant because ON length is typically only minimally sufficient to permit normal eye movement. In healthy adults, MRI in supine position has demonstrated that the ON is often only a few percent longer than the geometrically minimum distance from the orbital apex to the sclera in central gaze, and it becomes taut and stretched in adduction where the asymmetrically nasal attachment of the ON to the eye requires a longer path.^16^^–^^20^ As a result, the healthy ON elongates 0.4 ± 0.5 mm from central gaze to small adduction and 0.4 ± 0.5 mm further from small to larger adduction,^4^ although the glaucomatous ON does not stretch.^4^^,^^19^ Due to geometry, axial myopia requires additional ON elongation during eye movement. In the supine position, MRI has demonstrated that for each mm increase in globe axial length, ON elongation in large adduction increases by 0.2 mm^4^. Continuum mechanics has demonstrated another geometrical challenge arising from adduction with a length-limited ON: when the ON becomes taut in adduction, it shifts from behavior resembling a cantilever hinged at the resilient orbital apex to that of a truss transmitting high stress at the optic disc.^16^

The present findings suggest that ON loading on the globe during horizontal eye movements would be greater in lateral decubitus position than in supine position, particularly for the lower eye that translates directly along the anteroposterior path of the ON. This would suggest, for example, that horizontal eye rotations during sleeping or on-side video viewing would likely tether the globe more, and at smaller adduction angles, than in supine position. Because eye movements occur about once every two seconds during the rapid eye movement phase of sleep,^21^ globe translation in the lateral decubitus position may have clinical implications for sleep posture for people with ON vulnerabilities such as open angle glaucoma. The current findings suggest that supine posture would minimize ON traction caused by eye movements during sleep. However, the hypothetical benefit to the ON due to supine posture might be offset by other physiological effects of supine posture, including a greater risk of obstructive sleep apnea^22^ or cardiovascular changes.^23^ Additional studies are warranted before general clinical recommendations could be justified on the basis of the present study.

Although no MRI data are available on globe position in the upright position, the direction of gravity relative to the orbit is also orthogonal to the anatomical anteroposterior direction. Horizontal saccadic velocity is significantly lower than upright posture in 90° decubitus head tilts for both the higher and lower eye.^24^ It can be reasonably speculated that the eyes would probably translate anteriorly and perhaps slightly inferiorly, when changing posture from supine to upright, and that translation would probably be in the range of a little more than 1 mm. In microgravity as encountered during spaceflight, it is likely that both globes would translate anteriorly in the same direction, as there would be no asymmetrical gravitational influence to cause differential hydrostatic pressures in the orbits. Of course, in a terrestrial environment, hydrostatic pressure in the orbit would likely be related to central venous pressure and the level of the orbit relative to the heart.

Beyond intraocular hydrostatic pressure, extraocular muscle tension, and traction by the ON during eye rotations, the current findings reveal gravitational acceleration as yet another force acting physiologically on the eye. Gravity influences, and probably significantly so, the behavior of the eye relative to its surrounding orbital tissues in terrestrial and microgravity environments. This insight deserves consideration in future studies of behavior of ocular function in normal and pathological situations.
